# The Peripheral Blood Neutrophil-To-Lymphocyte Ratio Is Superior to the Lymphocyte-To-Monocyte Ratio for Predicting the Long-Term Survival of Triple-Negative Breast Cancer Patients

**DOI:** 10.1371/journal.pone.0143061

**Published:** 2015-11-18

**Authors:** Weijuan Jia, Jiannan Wu, Haixia Jia, Yaping Yang, Xiaolan Zhang, Kai Chen, Fengxi Su

**Affiliations:** 1 Guangdong Provincial Key Laboratory of Malignant Tumor Epigenetics and Gene Regulation, Sun Yat-Sen Memorial Hospital, Sun Yat-Sen University, Guangzhou, China; 2 Department of Breast Surgery, Sun Yat-Sen Memorial Hospital, Sun-Yat-Sen University, Guangzhou, China; 3 Department of breast surgery, The Second Affiliated Hospital of Guangzhou Medical University, Guangzhou, China; Sudbury Regional Hospital, CANADA

## Abstract

**Purpose:**

The peripheral hematologic parameters of patients can be prognostic for many malignant tumors, including breast cancer, although their value has not been investigated among the different molecular subtypes of breast cancer. The purpose of this study was to examine the prognostic significance of the neutrophil-to-lymphocyte ratio (NLR) and the lymphocyte-to-monocyte ratio (LMR) in different molecular subtypes of breast cancer.

**Methods:**

A retrospective cohort of 1570 operable breast cancer patients was recruited between January 2000 and December 2010. The counts of peripheral neutrophils, lymphocytes, monocytes and platelets were collected and applied to calculate the NLR and the LMR. Univariate and multivariate Cox proportional hazard analyses were used to assess the relationship of the NLR and the LMR with disease-free survival (DFS) and overall survival (OS) in all patients and triple negative breast cancer (TNBC) patients.

**Results:**

Univariate analysis revealed that lower NLR (≤2.0) and higher LMR (>4.8) were significantly associated with superior DFS in all patients (NLR, P = 0.005; LMR, P = 0.041) and in TNBC patients (NLR, p = 0.007; LMR, P = 0.011). However, multivariate analysis revealed that only lower NLR was a significant independent predictor of superior DFS and OS in all breast cancer patients (DFS, HR = 1.50 95% CI: 1.14–1.97, P = 0.004; OS, HR = 1.63, 95% CI: 1.07–2.49, P = 0.022) and in TNBC patients (DFS, HR = 2.58, 95% CI: 1.23–5.42, P = 0.012; OS, HR = 3.05, 95% CI: 1.08–8.61, P = 0.035). Both univariate and multivariate analysis revealed that neither the NLR nor the LMR significantly predicted DFS and OS among the patients with other molecular subtypes of breast cancer.

**Conclusions:**

A higher pretreatment peripheral NLR significantly and independently indicated a poor prognosis for breast cancer and TNBC, and this measurement exhibited greater prognostic value than a lower LMR. The NLR was not a prognostic factor for other breast cancer subtypes.

## Introduction

Breast cancer is currently the most common cancer among women, and the incidence of breast cancer increases yearly in China. Although breast cancer mortality rates have decreased worldwide in recent decades due to improvements in cancer treatment, breast cancer remains one of the leading causes of cancer death among females [1,2]. Several factors affect the prognosis of the breast cancer, including clinicopathological features (such as patient age, lymph node status, tumor size, etc.) and molecular biology parameters (such as hormonal receptors, human epidermal growth factor receptor 2 (HER2) and molecular subtype) [3].

It is widely realized that the prognosis of cancer patients depends on both tumor characteristics and patient-related factors. In recent decades, many studies have focused on inflammation and determined that cancer-related inflammation plays an important role in the development and prognosis of cancer. The host response in systemic inflammation has been considered an independent prognosis factor of cancer patients [4]. Furthermore, the relationship between inflammatory cells in the peripheral blood and cancer has garnered increased attention due to increasing evidence that the systemic inflammatory response is associated with alterations of peripheral blood white blood cells[5]. Moreover, peripheral blood cell tests are routinely performed for patients with cancer. It is easy to determine the severity of the systemic inflammatory response in patients with cancer via this simple test [5].

Recently, an increasing body of evidence has confirmed the utility of peripheral blood tests in predicting patient prognosis. Researchers have suggested that the hematologic components of the systemic inflammatory response may determine the prognoses of patients with cancer. In particularly, the pretreatment levels of peripheral neutrophils and leukocytes are independently predictors of survival in patients with cancer. Indeed, some recent studies have reported the predictive role of the neutrophil-to-lymphocyte ratio (NLR), the lymphocyte-to-monocyte ratio (LMR) and the platelet-to-lymphocyte ratio (PLR) for prognosis in a variety of cancers [5–9].

More recently, several studies have demonstrated the relationship between a high NLR and increased mortality in breast cancer [10]. Furthermore, Ni et al have reported that an elevated LMR is a favorable prognostic factor for breast cancer patients following neoadjuvant chemotherapy [11]. However, to our knowledge, no previous study has evaluated the NLR and the LMR in different molecular subtypes of breast cancer. The present study is the first large-scale cohort study to investigate the prognostic value of the peripheral blood NLR and LMR in breast cancer.

## Methods

Between January 2000 and December 2010, 1806 stage I-III breast cancer patients treated at Sun Yat-sen Memorial Hospital were recruited to the study. The patients’ data were obtained through electronic medical records, which were filled by a breast surgeon. In order to ensure the data quality, we assigned one person to collect the data, another one to check and confirm. A retrospective review of such patients was undertaken for the purpose of evaluating the predictive value of NLR and LMR for long-term survival in breast cancer. Of these patients, 1570 met the study inclusion criteria; data from a complete blood count and a leukocyte differential count before initiating any treatment and the medical records were available for these patients. Patients who had received any treatment before surgery or neoadjuvant chemotherapy or who had metastatic disease were not eligible for this study. Other exclusion criteria included bilateral breast cancer, male breast cancer, inflammatory breast cancer, and those on long term corticosteroids therapy, etc.

A blood analyzer (Sysmex [TOA Medical Electronics, Kobe, Japan]) was used to test all venous blood samples for the determination of the complete blood cell counts and the leukocyte differential counts. The neutrophil and lymphocyte counts were recorded, and the NLR and the LMR were calculated.

Estrogen receptor (ER), progesterone receptor (PR), and HER2 analyses were performed via immunohistochemistry (IHC). Tumors exhibiting greater than or equal to 10% positivity for ER or PR at any staining intensity among the total tumor cells were considered positive. The HER2 staining intensity score was evaluated from 0 to 3+ relative to the provided control slides, and specimens scored as 3+ or confirmed to display amplification based on florescence in situ hybridization were considered positive. The patients were categorized based on the IHC of their tumor in the following manner: luminal subtype: ER+ and/or PR+ and HER2-; HER2-positive subtype: HER2+; and triple-negative subtype: ER-, PR-, and HER2-.

Overall survival was calculated from the date of pathology diagnosis to death (of any causes) or the date of the last follow-up (First July 2014). Disease-free survival was calculated from the date of pathology diagnosis to the date of the local or distant recurrence, death, or new primary cancer. New primary breast cancer of the contralateral breast, tumors in other sites than breast, new in situ cancers were also considered as an event in DFS analysis. All patients were followed up every 3 months for the first 3 years and then every 1 year until relapse or death. The last follow-up date was first July 2014 for all of the available patients.

The ethics boards of Sun Yat-sen Memorial Hospital approved this retrospective study and waived the need for written informed consent from these patients. The patient data were anonymized throughout the study.

## Statistical Analysis

We performed receiver operating characteristic (ROC) curve analysis of the 5-year disease-free survival (DFS) to select the optimal threshold values of the NLR and used the LMR and the PLR to stratify the patients. The score at the point with a maximum Youden index (Youden index = sensitivity+specificity-1) was selected as the optimal threshold value.

The threshold values of the LMR and the NLR for DFS were 4.8 (≤4.8 vs >4.8) and 2.0 (≤2.0 vs >2.0), respectively.

The distributions of the continuous and categorical variables are displayed as the means and standard deviations and as the frequencies and percentages, respectively. To compare patients’ demographic and pre-treatment peripheral blood cells, the X^2^ test and T-test were used for categorical variables and continuous variables, respectively. The Kaplan-Meier method and the log-rank test were applied to analyze and compare DFS and overall survival (OS).

Univariate Cox regression models were used to investigate the relationship between each variable (including age, tumor size, lymph node status, molecular subtype, NLR, and LMR) and survival. Multivariate Cox proportional hazard models were applied to analyze independence, significance, and hazard discrimination. A P value less than 0.05 was considered statistically significant. The counting software SPSS (version 16.0, SPSS, Inc., Chicago, IL, USA) was applied for the above analysis.

## Results

The mean counts of white blood cells, neutrophils, monocytes and platelets were 6.71x10^9^ cells/L (range, 2.34–15.76x10^9^ cells/L), 4.11x10^9^ cells/L (range, 0.93–12.11x10^9^ cells/L), 0.44x10^9^ cells/L (range, 0.01–2.39x10^9^ cells/L), and 242.23 cells/L (range, 50–528 cells/L), respectively. The median NLR and LMR were 2.0 (range, 0.4–15.0) and 4.7 (range, 0.6–201), respectively. ROC curve analysis was performed to determine the optimal threshold values of the NLR (AUC = 0.540), the LMR (AUC = 0.556) and the PLR. However, we did not identify a significant threshold value of the PLR. The NLR and LMR thresholds for 5-year DFS were 2.0 and 4.8, respectively (S1 Fig). All patients were grouped into the high (>2.0) and low NLR (≤2.0) groups or into the high (>4.8) and low LMR (≤4.8) groups. When all patients were grouped according to the NLR or the LMR, differences in DFS and OS were observed between the groups. There was no difference between groups in patients undergoing chemotherapy (Table 1).

**Table 1:** Baseline characteristics of the patients according to the NLR and the LMR.

Variable	N	NLR		P value	LMR		P value
		>2 (%)	≤2 (%)		>4.8 (%)	≤4.8 (%)	
Age (years)	1570	49.6 ±12.4	48.1±11.1	0.015	48.2 ± 12.0	49.6 ±11.6	0.027
Tumor size	1468			0.322			0.065
T1		440 (59.0)	413 (57.2)		439 (57.5)	414 (58.7)	
T2		260 (34.9)	250 (34.6)		258 (33.80	252 (35.7)	
T3-T4		46 (6.2)	59 (8.2)		66 (8.70	39 (5.5)	
Tumor grade	1393			0.884			0.441
G1		51 (7.2)	51 (7.5)		47 (6.6)	55 (8.1)	
G2		393 (55.2)	367 (53.9)		399 (55.8)	361 (53.2)	
G3		268 (37.6)	263 (38.6)		269 (37.6)	262 (38.6)	
Lymph node status	1491			0.818			0.970
Negative		439 (57.7)	426 (58.3)		442 (57.9)	422 (58.0)	
Positive		322 (42.3)	305 (41.7)		321 (42.1)	306 (42.0)	
Molecular subtype	1570			0.160			0.882
Luminal		524 (65.2)	477 (62.3)		518 (63.9)	483 (63.6)	
HER2-positive		178 (22.1)	166 (21.70		180 (22.2)	164 (21.60	
Triple-negative		102 (12.7)	123 (16.1)		113 (13.9)	112 (14.8)	
Chemotherapy	1570			0.125			0.069
No		128 (15.9)	101 (13.2)		131 (16.2)	98 (12.9)	
Yes		676 (84.1)	665 (86.8)		680 (83.8)	661 (87.1)	
WBC (10^9^ cells/L)	1570	6.2 ± 1.6	7.2 ± 1.9	<0.001	6.8 ± 1.9	6.6 ± 1.8	0.085
Intraoperative NSAID	1560			0.139			0.002
Yes		530 (70.0)	585 (30.0)		567 (80.0)	548 (70.0)	
No		230 (30.0)	215 (30.0)		187 (20.0)	258 (30.0)	
Neutrophil count (10^9^ cells/L)	1570	3.3 ± 1.0	4.9 ± 1.6	<0.001	4.3 ± 1.6	3.9 ± 1.4	<0.001
Lymphocyte count (10^9^ cells/L)	1570	2.3 ± 0.6	1.7 ± 0.5	<0.001	1.7 ± 0.5	2.3 ± 0.7	<0.001
Monocyte count (10^9^ cells/L)	1570	0.6 ± 4.0	0.6 ± 4.9	0.793	0.8 ± 6.2	0.4 ± 0.1	0.027
Platelet count (10^9^ cells/L)	1570	237.2 ± 60.3	247.5 ± 62.0	<0.001	241.6 ± 62.8	242.9 ± 59.8	0.666
10-year DFS (95% CI)	1570	77.72 (73.82–81.62)	84.07 (80.77–87.38)		91.59 (88.33–94.86)	87.64 (84.43–90.86)	
10-year OS (95% CI)	1570	87.53 (84.04–91.01)	91.39 (88.37–94.40)		82.59 (78.63–86.55)	79.31 (75.89–82.73)	

The baseline characteristics of the breast cancer patients are summarized according to the NLR and LMR values in Table 1. All of the patients were female. The patients ranged in age from 23 to 91 years, and the median age was 47 years at the time of diagnosis. The majority of patients (87.3%) were classified as stage T1-T2, and more than half of the patients (58.0%) were free of axillary lymph node metastases. More than half of the patients (56.0%) underwent a modified radical mastectomy. Most patients (85.4%) received chemotherapy. 1115/1560 patients received flurbiprofen axetil at the end of the surgery, and 232 of 1115 also received parecoxib at the beginning of the surgery.

After a median follow-up of 79 months (range, 4–172 months), 242 patients experienced relapse, and 108 patients had died. The 5-year DFS and OS rates were 85.7% and 94.6%, respectively. The 10-year DFS and OS rates were 80.9% and 89.5%, respectively. In the univariate analysis, the factors related to the prognosis of patients with breast cancer included age, T stage, N stage, tumor grade, molecular subtype, NLR status and LMR status. Especially in all patients, a high NLR and a low LMR were significant predictors of disease recurrence and mortality compared with a low NLR and a high LMR, respectively (S1 Table). The multivariate COX regression model analysis controlled for all factors with significant associations emerging from the univariate analysis. Application of this model revealed that NLR remained an independent predictor of DFS (HR = 1.50, P = 0.004) and OS (HR = 1.63, P = 0.022) in all patients (S1 Table). We also investigated the distribution of the hematological parameters among different molecular subtypes of breast cancer. The NLR was higher in triple-negative breast cancer (TNBC) than in the other subtypes (P = 0.032) (S2 Table).

In univariate analysis, no significant change in the rate of cancer recurrence and death were found in the patients who received preoperative parecoxib, postoperative flurbiprofen, and no NSAIDs (S1 Table). Univariate analysis also showed that intraoperative administration of NSAIDs was not correlated with DFS and OS in luminal subtype (DFS: HR = 0.81 (0.56–1.18) P = 0.273; OS: HR = 1.04 (0.55–1.94) p = 0.912) and HER2 enriched subtype (DFS: HR = 1.03 (0.63–1.61) P = 0.990; OS: HR = 0.93 (0.51–1.85) p = 0.932). A statistical analysis of NSAIDs and recurrence of TNBC was impossible, because there were only 9 triple negative breast cancer patients who had not received NSAIDs.

**Fig 1:**
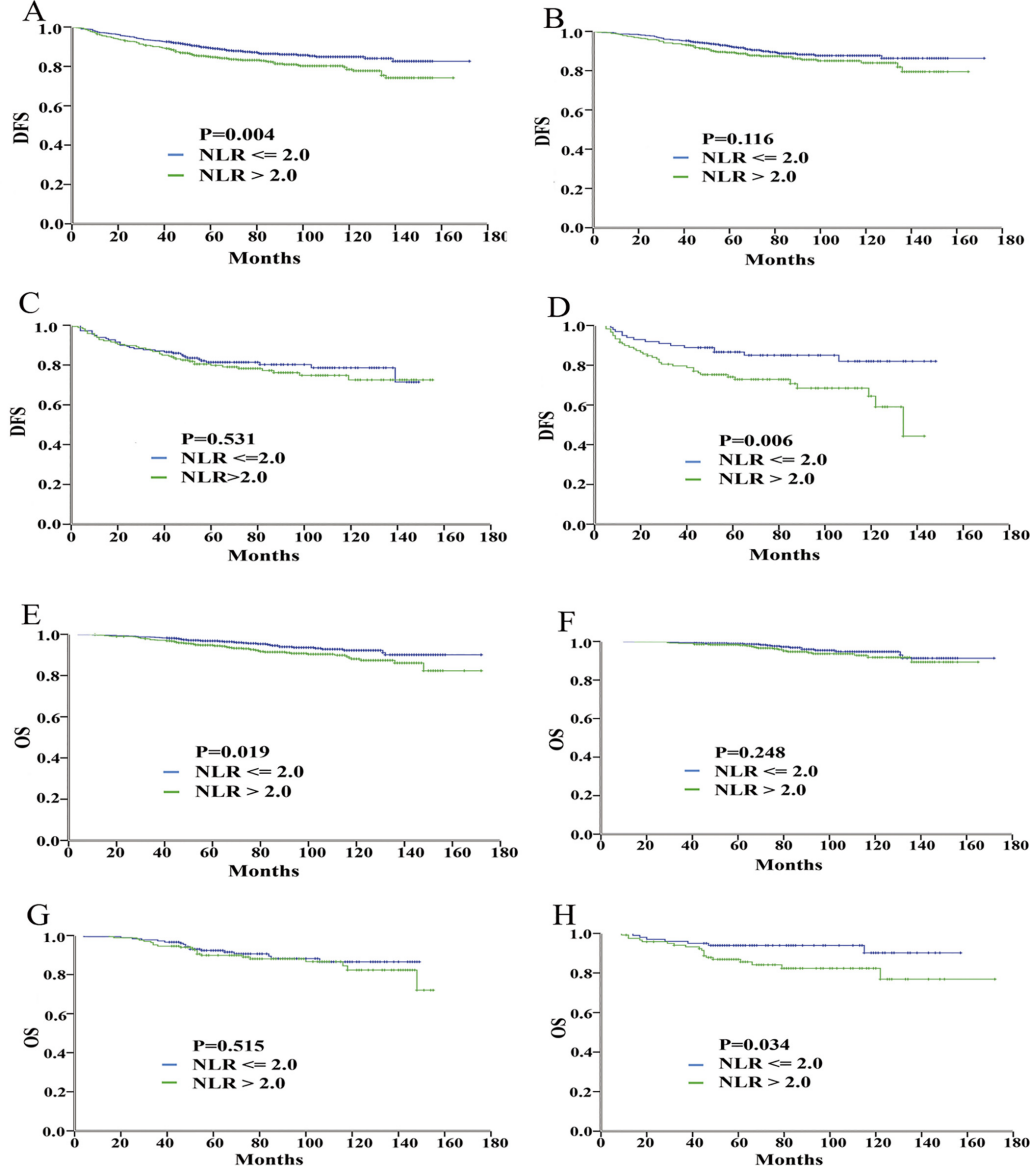
Prognostic value of NLR for breast cancer patients. Kaplan-Meier curves of DFS for all patients (A), DFS for the patients with ER-positive and/or PR-positive/HER2-negative disease (B), DFS for the patients with HER2-positive disease (C), DFS for the patients with ER-negative/PR-negative/HER2-negative disease (D), OS for all patients (E), OS for the patients with ER-positive and/or PR-positive/HER2-negative disease (F), OS for the patients with HER2-positive disease (G), and OS for the patients with ER-negative/PR-negative/HER2-negative disease (H).

**Fig 2:**
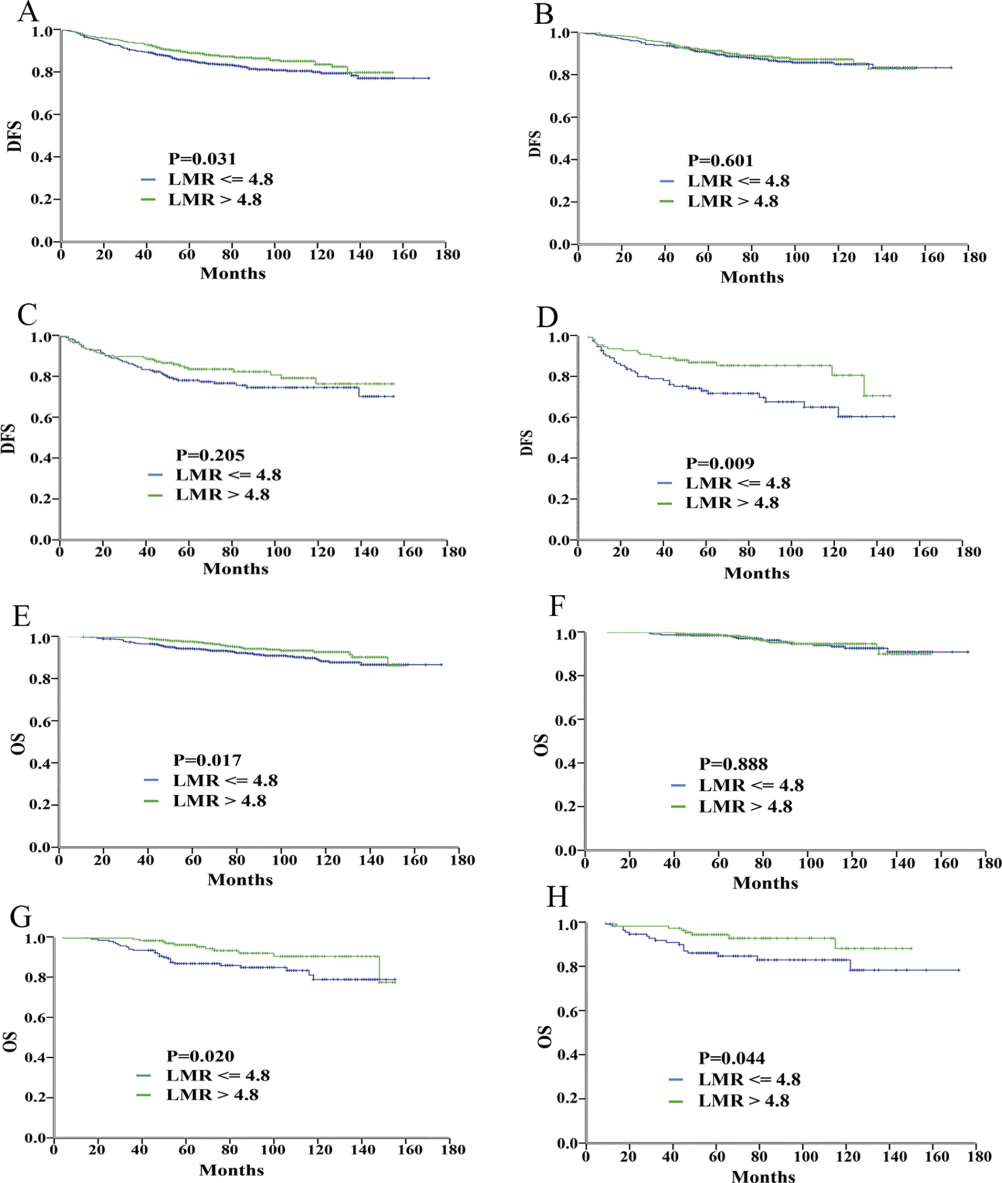
Prognostic value of the LMR for breast cancer patients. Kaplan-Meier curves of DFS for all patients (A), DFS for the patients with ER-positive and/or PR-positive/HER2-negative disease (B), DFS for the patients with HER2-positive disease (C), DFS for the patients with ER-negative/PR-negative/HER2-negative disease (D), OS for all patients (E), OS for the patients with ER-positive and/or PR-positive/HER2-negative disease (F), OS for the patients with HER2-positive disease (G), and OS for the patients with ER-negative/PR-negative/HER2-negative disease (H).

Kaplan-Meier curves of DFS and of OS for all breast cancer subtypes according to the NLR and the LMR thresholds are presented in Figs 1 and 2, respectively. Notably, for the TNBC patients with a high and low NLR, the 10-year DFS was 58.4% and 82.1%, respectively (HR = 2.28; 95% CI: 1.25–4.18), and the 10-year OS was 80.0% and 90.2%, respectively (HR = 2.47; 95% CI: 1.04–5.89). Similarly, for the TNBC patients with a low and high LMR, the 10-year DFS was 58.6% and 81.3%, respectively (HR = 2.14; 95% CI, 1.19–3.83), and the 10-year OS was 77.0% and 88.5%, respectively (HR = 2.30; 95% CI, 1.00–5.29). In contrast, no significant difference in the 10-year outcomes was detected based on the NLR or the LMR for the other two subtypes of breast cancer patients.

Based on above analysis, high NLR and low LMR were significantly associated with a lower DFS rate and higher mortality only in TNBC. No significant differences in survival according to the NLR or the LMR values were observed for the other breast cancer subtypes (Figs 1 and 2; Table 2). We used both univariate and multivariate statistical techniques to investigate the association of age, tumor size, node status, blood cells, NLR status and LMR status and the prognosis of TNBC patients. Univariate analysis revealed that age, node status, NLR status and LMR status were prognostic factors for survival of TNBC. Multivariate analysis revealed that the NLR was the only independent prognostic factor for DFS (HR = 2.58, P = 0.012) and OS (HR = 3.05, P = 0.035) among the TNBC patients (Table 2).

**Table 2:** DFS and OS of the TNBC patients based on univariate and multivariate Cox proportional regression analyses.

	Univariate				Multivariate			
	DFS		OS		DFS		OS	
	Hazard ratio (95% CI)	P value	Hazard ratio (95% CI)	P value	Hazard ratio (95% CI)	P value	Hazard ratio (95% CI)	P value
Age	1.03 (1.01–1.06)	0.003	1.07 (1.04–1.10)	<0.001	1.05 (1.02–1.08)	<0.001	1.09 (1.05–1.13)	<0.001
Nodal status (positive vs negative)	2.46 (1.37–4.42)	0.003	2.57 (1.09–6.03)	0.030	2.24 (1.17–4.28)	0.015	2.45 (1.00–6.14)	0.055
T2 (vs T1)	1.53 (0.82–2.88)	0.185	1.83 (0.79–4.23)	0.157	1.35 (0.69–2.67)	0.383	1.49 (0.59–3.74)	0.401
T3 (vs T1)	1.61 (0.55–4.70)	0.386	0.68 (0.09–5.32)	0.717	1.62 (0.54–4.88)	0.395	0.65 (0.08–5.26)	0.687
NLR>2 (vs ≤2)	2.28 (1.25–4.18)	0.007	2.47 (1.04–5.89)	0.041	2.58 (1.23–5.42)	0.012	3.05 (1.08–8.61)	0.035
LMR≤4.8 (vs >4.8)	2.14 (1.19–3.83)	0.011	2.30 (1.00–5.29)	0.050	1.47 (0.75–2.92)	0.265	1.33 (0.52–3.45)	0.554
WBC	1.12 (1.00–1.29)	0.101	1.13 (0.93–1.372)	0.218	/	/	/	/
PLT	1.00 (1.00–1.00)	0.671	1.00 (0.99–1.01)	0.995	/	/	/	/
Lymphocytes	0.91 (0.60–1.40)	0.664	0.98 (0.55–1.76)	0.946	/	/	/	/
Neutrophils	1.19 (1.00–1.42)	0.054	1.18 (0.92–1.51)	0.185	/	/	/	/
Monocytes	2.07(0.57–7.52)	0.269	3.22 (0.66–15.65)	0.147	/	/	/	/

## Discussion

It has increasingly been recognized cancer is a systemic, not local, disease. Indeed, breast cancer presents as a spectrum of malignant disorders affecting the same organ [12]. Clinical features and molecular pathology are the main determinants of current strategies and predictions of the prognosis of breast cancer. However, these biomarkers are far from meeting the medical need. Additional biomarkers are needed to direct therapy and judge prognosis [13,14].

The relationship between the inflammatory system and cancer has been demonstrated by accumulating studies. The pretreatment counts of peripheral inflammatory cells, including neutrophils, lymphocytes and monocytes, have demonstrated the strong link between the inflammatory system and prognosis in different types of cancer[15]. In particular, NLR and LMR have recently been reported to be prognostic factors in several types of cancers [7,10,16]. Here, we performed a large-scale cohort study of breast cancer patients to evaluate the prognostic value of the peripheral neutrophil, lymphocyte, monocyte counts, NLR and LMR, as well as other clinical factors.

In this study, the optimal thresholds of 2.0 for the NLR and of 4.8 for the LMR were found to exhibit superior prognostic value for the DFS of breast cancer. However, we did not detect any threshold for the PLR, possibly due to the larger variation in platelet counts. This study used different NLR and LMR thresholds from previous studies, which may hinder the comparability of their results with those of our study. The primary reason for this difference was the number of breast cancer patients from different geographic regions and races. Other reasons included the different survival endpoints used to determine the threshold of NLR. DFS was used as the endpoint to assess the prognostic value of the NLR in our study, whereas OS was used in other studies [10].

Azab et al first reported that NLR was a predictor of mortality in breast cancer. [10]. In their study, the NLR before chemotherapy predicted short- and long-term mortality and correlated with tumor size and patient age. Based on our results, the NLR was associated with DFS and mortality independently of tumor size and patient age. Our results also demonstrated that patients with higher NLR and lower LMR values were younger than patients with lower NLR and higher LMR values. This finding was consistent with the findings of another recent study [17]

Nonetheless, the present study is the first study to evaluate the prognostic value of both the NLR and the LMR in breast cancer subtypes. Our study demonstrated that pretreatment NLR is an independent, significant predictor of long-term DFS and OS among breast cancer patients. However, multivariate analysis did not demonstrate any prognostic significance of LMR for DFS or OS, likely because the strong predictive ability of NLR affected the predicative function of LMR on multivariate analysis. Thus, NLR was superior to LMR in the prognosis of breast cancer.

Over the past decade, microarray-based gene expression studies have demonstrated that breast cancer comprises a heterogeneous group of diseases that exhibit different distinct molecular features [18]. Our study also focused on the NLR and the LMR and their prognostic values within each breast cancer subtype. Elevated NLR and decreased LMR were significantly associated with poor prognosis for the TNBC subtype. However, based on multivariate analysis, only the NLR remained an independent significant predictor of DFS and OS in TNBC patients. TNBC is well known to exhibit poor clinical outcomes compared with non-TNBC and is a hot issue in present breast cancer research. Increasing numbers of studies have suggested that the TNBC subtype may be more strongly influenced by systemic inflammatory function. The tumor immune response plays an important role in the pathobiology of TNBC, and the clinical trials examining treatment with immunomodulating agents for TNBC are ongoing [19]. Retsky et al reported that the perioperative administration of the nonsteroidal anti-inflammatory drug ketorolac can reduces relapses of breast cancer by five-fold in the 9–18 month period after surgery, and triple-negative breast cancer may be the ideal group in which to apply this treatment [20]. Tumor-associated lymphocytes (TILs) have been reported to be more frequent in TNBC samples than in hormone receptor-positive breast cancer and as an independent prognostic marker of DFS and OS in TNBC [21,22]. Engel et al found that TNBC cells stimulate a significantly stronger natural killer cell immune response than ER-positive breast cancer cells and that the infiltration of immunosuppressive Tregs (CD4+ T-cells, CD8+ T-cells, and forkhead box P3-positive (Foxp3) regulatory T-cells) was increased in human TNBC specimens [23]. High Foxp3+TIL levels and a high total number of CD8+ TILs was strongly associated with improved survival in TNBC [19]. Therefore, TILs at diagnosis likely indicate an ongoing antitumor immune response [24,25]. TNBC is a poorly differentiated tumor subtype and, therefore, may contain more antigenic tumor variants than the other HER2-negative breast cancer subtypes [22,26].

Our findings are consistent with the results of the study of Yao et al, who reported that the NLR was an independent predictor of mortality in TNBC, and they used a cutoff value of NLR determined by the ROC curve of DFS at 2 years, but there was no statistically significant difference in tumor relapse in TNBC when patients were stratified by NLR [27]. Another study focused only on the luminal A subtype, and the authors found that the NLR was an independent prognostic factor for the luminal A breast cancer subtype [28]. The reasons for these inconsistent results are various, including the following possible reasons: First, these studies were retrospective. Second, the sample size in these studies was small, and the sample size of TNBC patients was even smaller. Finally, the follow-up duration in these studies was short.

The association of LMR with cancer survival has been reported for several cancers. Higher LMR levels are associated with favorable DFS of breast cancer patient under neoadjuvant chemotherapy [7,11,29]. Although the LMR predicted DFS and OS in all patients and in the TNBC patients in this study, the NLR was shown to outperform the LMR. Univariate analyses revealed that the correlation between the NLR and patient survival was much more significant than that between the LMR and patient survival, and multivariate analyses found that NLR was the only independent predictor of DFS and OS in TNBC.

The cut-off value for both ER and PR positive was ≥10% for IHC (recommended by the European guidelines [30]) in our laboratory before 2007. In order to keep the data consistency, we used the same cut-off value. However, this limitation had a little impact on our results for the following two reasons. First, some studies have tested that the vast majority of <10% IHC ER-positive tumors showed molecular features similar to those of ER-negative, basal-like molecular characteristics [31–33]. Second, some studies found that a few tumors have an ER expression level ranging between 1% and 9% [34]. In our study only a small proportion of patients (4/758) with tumors that has 1–9% positive ER or PR.

In this cohort, we did not observe the association between intraoperative use of NSAIDs and the outcome of breast cancer. There could be following reasons through analysis: First, Some study found that the intraoperative use of ketorolac or diclofenac was associated with an improved survival, but other analgesics were not associated with a reduction in cancer recurrence rates[17,35,36]. In our study, the intraoperative analgesics included flurbiprofen axetil and parecoxib, without ketorolac or diclofenac. The use of different NSAIDs drugs might be a reason of the negative results. Second, Forget et al reported that NSAIDs use at the beginning of the surgery was associated with a lower recurrence of lung cancer[17]. However, in this study, almost all patients received NSAIDs at the end of the surgery. The use of NSAIDs at the beginning of the surgery may be more effective than at the end of the surgery. Third, Retsky et al suggested that NSAIDs used perioperatively might likely reduce the recurrence of TNBC, rather than the recurrence of luminal and HER2 enriched subtypes[20]. Furthermore, it is possible that all the above mentioned factors could act together. Although NSAIDs are common analgesics used perioperatively, they may be related to potential adverse effects, such as fatal cardiovascular events, gastrointestinal bleeding, colorectal anastomotic leakage, surgical bleeding, renal impairment, and mpaired fracture healing. The studies of perioperative use of NSAIDs demonstrated controversial results[37]. In this cohort, the major side effects associated with NSAIDs was haemorrhage. Nevertheless, there was no significant difference between patients who did or did not receive NSAIDs and the development of a significant bleeding event. We did not observe any other NSAIDs-related side effects

The present study exhibits several advantages over previous studies. DFS was selected as the endpoint for the NLR, the PLR, and the LMR analyses, which may exclude the influence of cancer unrelated to death. In addition, this study included a relatively large sample size compared with other related studies. Moreover, the median follow-up duration was nearly 80 months in our study. However, there are three major limitations to this study. This study is a single-center retrospective study. Lack of pre-existing listing or prospective database is one of the major limitations of our study. Although, we had identified all of the patients that fitted our inclusion criteria in our hospital, there were some other inherent biases in our study, such as, selection bias, uncontrolled and unrecognized biases. Furthermore, this study lacked an evaluation of tumor-associated neutrophils and lymphocytes. Moreover, the NLR can be affected by internal and external factors, including acute and chronic infection and lifestyle-related habits [38]. It was documented that NLR was elevated in patients after glucocorticoids exposure [39]. We were not able to abtain information about the detailed use of corticosteroids. However Lorente and colleagues found that NLR prognostic value was independent of prior use of corticosteroids [40]. Thus, further prospective multicenter studies are needed to determine the strengths and deficiencies of these results.

In conclusion, the findings of our study indicate that a preoperative elevated NLR is an independent prognostic biomarker for DFS and OS in TNBC that is superior to the LMR. These results suggest that cancer-associated inflammation may play a greater role in promoting breast cancer progression in TNBC than in other breast cancer subtypes. However, further validation studies are required.

## Supporting Information

S1 Dataset The raw data of 1570 primary breast cancer patients. (XLSX)

S1 Fig The ROC curves for 5-year DFS according to the NLR and the LMR. (TIF)

S1 Table DFS and OS of all patients using univariate and multivariate* COX proportional regression analysis. * COX regression with the forward stepwise method was used to do multivariate analyses.
(XLSX)

S2 Table The distribution of hematological parameters in different molecular subtypes. (XLSX)
