# Supplementary figures and images for: The Peripheral Blood Neutrophil-To-Lymphocyte Ratio Is Superior to the Lymphocyte-To-Monocyte Ratio for Predicting the Long-Term Survival of Triple-Negative Breast Cancer Patients
Source: PLoS One. 2015 Nov 18;10(11):e0143061. doi: 10.1371/journal.pone.0143061 (PMC4666347; doi:10.1371/journal.pone.0143061)

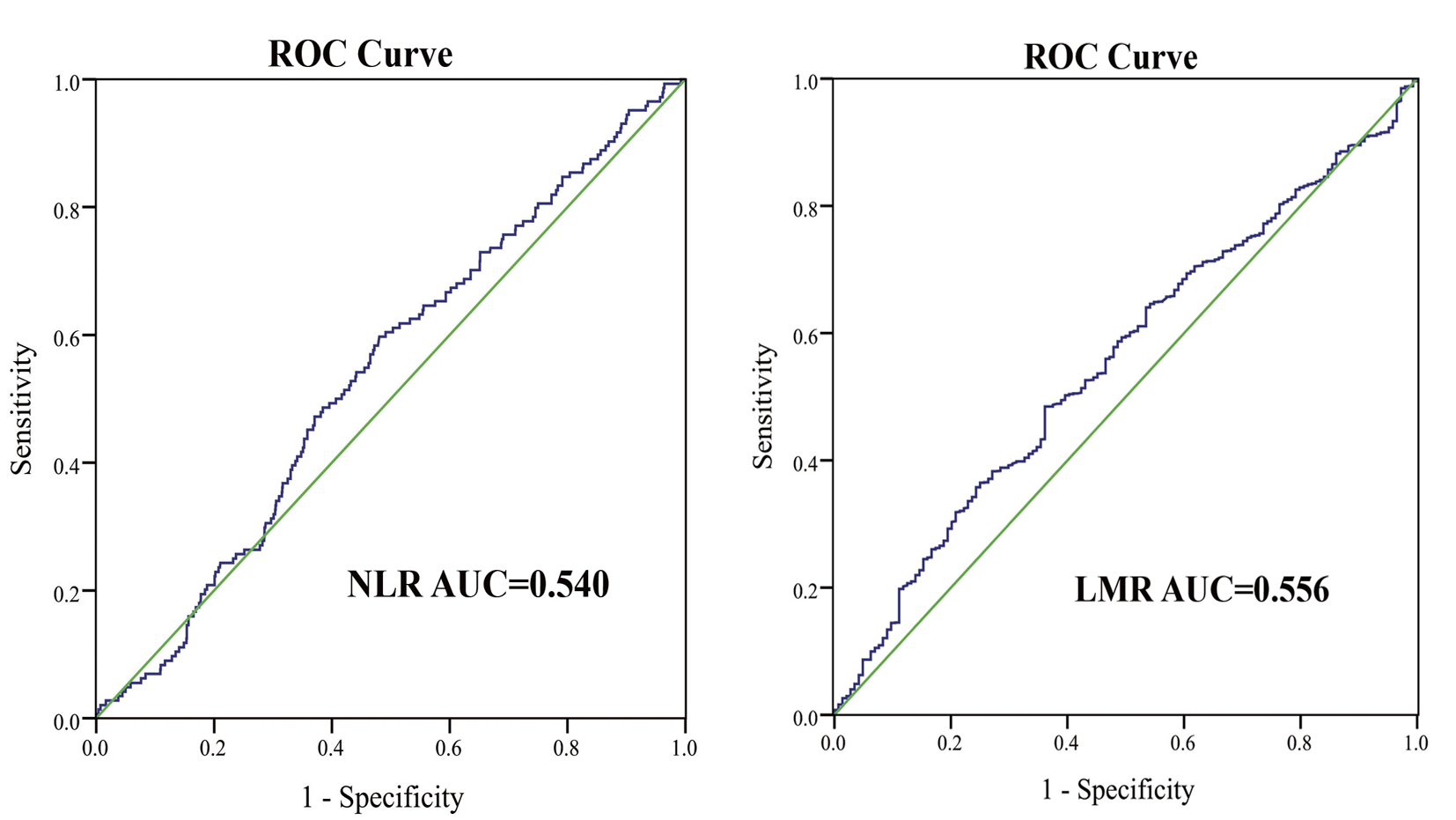

Supplement: S1 Fig — (TIF) [file pone.0143061.s002.TIF]
